# Level of macroautophagy drives senescent keratinocytes into cell death or neoplastic evasion

**DOI:** 10.1038/cddis.2014.533

**Published:** 2014-12-18

**Authors:** E Deruy, J Nassour, N Martin, C Vercamer, N Malaquin, J Bertout, F Chelli, A Pourtier, O Pluquet, C Abbadie

**Affiliations:** 1 CNRS, UMR8161, Institut de Biologie de Lille, Lille F-59021, France; 2 University Lille 1 59650, Villeneuve d'Ascq, France; 3 University Lille 2, Lille 59000, France; 4 Institut Pasteur de Lille, Lille F-59000, France

## Abstract

Senescence is a non-proliferative state reached by normal cells in response to various stresses, including telomere uncapping, oxidative stress or oncogene activation. In previous reports, we have highlighted that senescent human epidermal keratinocytes have two opposite outcomes: either they die by autophagic programmed cell death or they evade in the form of neoplastic postsenescence emergent (PSNE) cells. Herein, we show that partially reducing macroautophagy in senescent keratinocytes using 3-methyl adenine or anti-Atg5 siRNAs increases the PSNE frequency, suggesting that senescent keratinocytes have to escape autophagic cell death to generate PSNE cells. However, totally inhibiting macroautophagy impairs PSNE and leads to a huge accumulation of oxidative damages, indicating that senescent keratinocytes need to achieve quality-control macroautophagy for PSNE to occur. In accordance, we demonstrate that the progenitors of PSNE cells display a level of macroautophagy slightly lower than that of the average senescent population, which is directly dictated by their level of reactive oxygen species, their level of upregulation of MnSOD, their level of activation of NF-*κ*B transcription factors and their level of dysfunctional mitochondria. Macroautophagy thus has antagonistic roles during senescence, inducing cell death or promoting neoplastic transformation, depending on its level of activation. Taken together, these data suggest that levels of oxidative damages and ensuing macroautophagic activity could be two main determinants of the very initial phases of neoplastic transformation by senescence evasion.

Initially described as the phase reached by human normal fibroblasts after a limited number of serial passages in culture,^1^ senescence is now recognized as a fundamental program that affects several cell properties. The senescence program includes a cell cycle arrest mediated by the p53/p21^WAF1^ and/or p16^INK4a^/pRB pathways,^2^ changes in chromatin organization,^3^ changes in transcriptome,^4^ proteome^5^ and secretome,^6, 7^ increase in cell volume^8, 9^ and increase in macroautophagic activity.^10, 11^ Senescent cells accumulate in tissues with aging and contribute to age-related pathologies.^12^

The senescence program is activated in response to various stresses, including telomere dysfunction,^13, 14^ irreparable DNA damage,^15^ oxidative stress^16^ or activation of Ras^17^ or NF-*κ*B.^18^ Oxidative damage seems to be the one common denominator of several senescence inducers. Indeed, chronological age,^19^ various stresses known to accelerate aging such as ionizing and ultraviolet (UV) radiations^16^ and activation of Ras and NF-*κ*B^18, 20^ were all associated with accumulation of reactive oxygen species (ROS). ROS attack to DNA results in a DNA-damage response which, by itself or in addition to that activated by telomere uncapping, leads to the typical cell cycle arrest encountered by senescent cells.^15^ Moreover, ROS damage all other organelles and macromolecules, what contributes notably to the increase in macroautophagic activity associated with senescence.^21^

Numerous data suggest that senescence corresponds to an irreversible growth arrest that cells have to bypass to become tumorigenic. However, this cell cycle arrest is not always irreversible, notably in epithelial cells that are at the origin of the most frequent cancers in human. We and others have shown that normal human epidermal keratinocytes (NHEKs)^22^ or human mammary epithelial cells (HMECs)^23^ having reached the senescence plateau, although displaying all the characteristics of senescent cells, spontaneously reactivate a mitotic process to generate postsenescence emergent cells, which are transformed and able to form skin hyperplasia or carcinoma in *nude* mice. Several data from our group suggest that the oxidative DNA damages encountered by senescent NHEKs could be the mutagenic motor of this postsenescence neoplastic emergence (PSNE).^22, 24^

Macroautophagy is a process enabling isolation of cellular components inside a specific double-membrane vesicle, the autophagosome, and their degradation after the autophagosome has fused with a lysosome.^25, 26^ The different steps of the process are orchestrated by >30 ATG genes.^27^ In brief, the starting of the process is under the control of ATG6/Beclin-1 and a class III PI3 kinase, hVps34.^28, 29^ The completion of the autophagosome formation and its fusion with lysosomes to form an autolysosome are driven by an ATG12–ATG5–ATG16 complex^30^ and by the integration of ATG8/LC3 in the autophagosome membrane.^31^ Macroautophagy was characterized in yeast as an in-bulk degradative pathway induced by nutrient deprivation. In that situation, it is assumed to non-selectively degrade cytosolic components and organelles to produce metabolites, which will be used to synthesize indispensable new components and generate energy, hence helping cells to survive.^32^ Although less well characterized, it is now clear that a basal constitutive macroautophagic activity also exists to contribute to selective disposal of misfolded aggregated proteins or altered organelles. This form of autophagy is often referred as housekeeping autophagy or quality-control autophagy.^33^ When cells are stressed, this autophagic activity is enhanced to help face damages and again survive. However, if it is overactivated or prolonged, it can lead to an opposed outcome, i.e., cell death, through the excessive elimination of vital cell proteins or organelles. This mechanism of cell death was shown to occur in various physiological and pathological situations, besides or instead of apoptosis. It is as such referred as type II programmed cell death (type I being apoptosis) or as autophagic programmed cell death.^25, 34, 35^

We have shown that senescent NHEKs experience an increase in macroautophagic activity whose excessive intensity leads to their death.^10^ Therefore, two antagonistic outcomes are possible for senescent keratinocytes: autophagic programmed cell death for most cells^10^ or mitotic activity recovery and PSNE for about 1 cell on 10 000.^22^ In the present report, we addressed the question of the role of macroautophagy in the senescence/PSNE balance. We show that among senescent keratinocytes, the progenitors of PSNE cells display an autophagic activity slightly lower than the average, what allows them to avoid autophagic cell death and to ensure the quality control indispensable for mitosis re-entry. This means that the outcome of senescent keratinocytes is dependent, at least in part, on their level of macroautophagic activity. We also investigated the relationship between the oxidative stress encountered by senescent keratinocytes, their level of autophagic activity and their final outcome. Indeed, we had previously shown that the lethal autophagic activity of senescent keratinocytes is induced following oxidative damages to mitochondria and nucleus.^21^ But we had also shown that oxidative stress is necessary and sufficient for PSNE, in correlation with the generation of mutagenic DNA damages, including DNA breaks and 8-oxo-guanines.^22^ Here we show that the probability of senescent cells to undergo PSNE is directly correlated to their macroautophagy, which is itself directly correlated to the activation of the NF-*κ*B/MnSOD/H_2_O_2_ pro-oxidant pathway. This suggests that the oxidative damages occurring during senescence and the way senescent cells face up to them using macroautophagy are important parameters of the earliest steps of carcinogenesis occurring by senescence evasion during aging.

## Results

**Figure 1:**
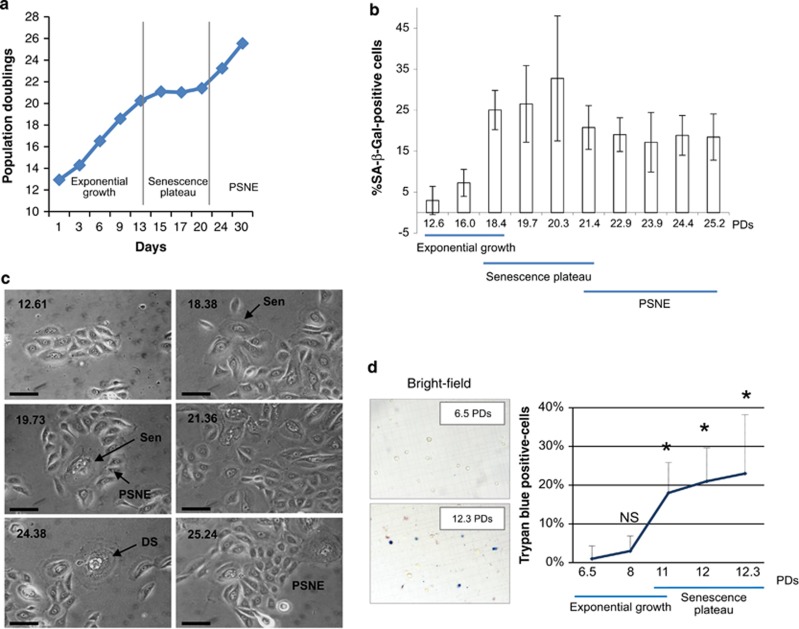
Growth curve and characteristics of *in vitro*-cultivated NHEKs. (**a**) Growth curve of NHEKs 4F0315 showing the exponential growth phase, the senescence plateau and the PSNE phase. (**b**) Percentage of SA-*β*-Gal-positive cells manually counted in 5–28 random fields comprising 20–262 cells. Note the senescence plateau between 18.4 and 20.3 population doublings, and PSNE from 21.4 doublings, during which some SA-*β*-Gal-positive senescent cells persist. Each bar represents mean±S.D. (**c**) Cell morphologies observed by phase-contrast microscopy. The doubling number is indicated on each photograph. At doubling 12.61, cells have the typical morphology of epithelial cells growing as islet. At doubling 18.38, most cells have increased in size and display some small vesicles. The image shown at doubling 19.73 illustrates a senescent cell, recognizable by its large size and the great number of vesicles, which has generated PSNE cells by a budding mitosis mechanism. At doubling 21.36, the culture dish comprises a mixed population of senescent and PSNE cells. From 24.38 doublings, senescent cells dying by autophagic cell death are observable among PSNE cells. Sen: senescent cell; DS: dying senescent cell. Bar represents 20 *μ*m. (**d**) Quantification of cell death by Trypan blue exclusion in exponentially growing (6.5 and 8 PDs) and senescent (11, 12 and 12.3 PDs) NHEKs K18FC. Non-viable cells (blue) were counted under the microscope in four independent hemocytometer chambers for a total of at least 100 cells. The results are presented as the mean±S.D. percentage of dead cells of all counts. Significant differences are indicated with *P<0.01. NS, not significant

All the experiments were performed with NHEKs stemming from healthy donors. NHEKs undergo an exponential growth phase and then reach a plateau (Figure 1a) at which they exhibit all the senescence markers, including increase in senescence-associated-*β*-galactosidase (SA-*β*-Gal) activity (Figure 1b), increase in cell size (Figure 1c) and growth arrest evidenced by PCNA downregulation and p16 and p21 upregulation.^22^ From this senescent stage, NHEKs experience two alternative outcomes. Either they massively die (Figure 1d and Gosselin *et al.*^10^) through an excessive autophagic activity^10, 21^ or, for a small fraction of cells (about 1 on 10 000), they undergo an atypical budding mitosis generating clones of PSNE cells that invade the culture dishes (Figure 1c and Gosselin *et al.*^22^). PSNE cells were shown by a transcriptomics analysis to display transformed characteristics and were evidenced to be able to generate some small skin hyperplasias and non-melanoma carcinomas in *nude* mice assays.^22, 24^

### Postsenescence neoplastic emergent cells have a lower macroautophagic activity than their senescent progenitors

**Figure 2:**
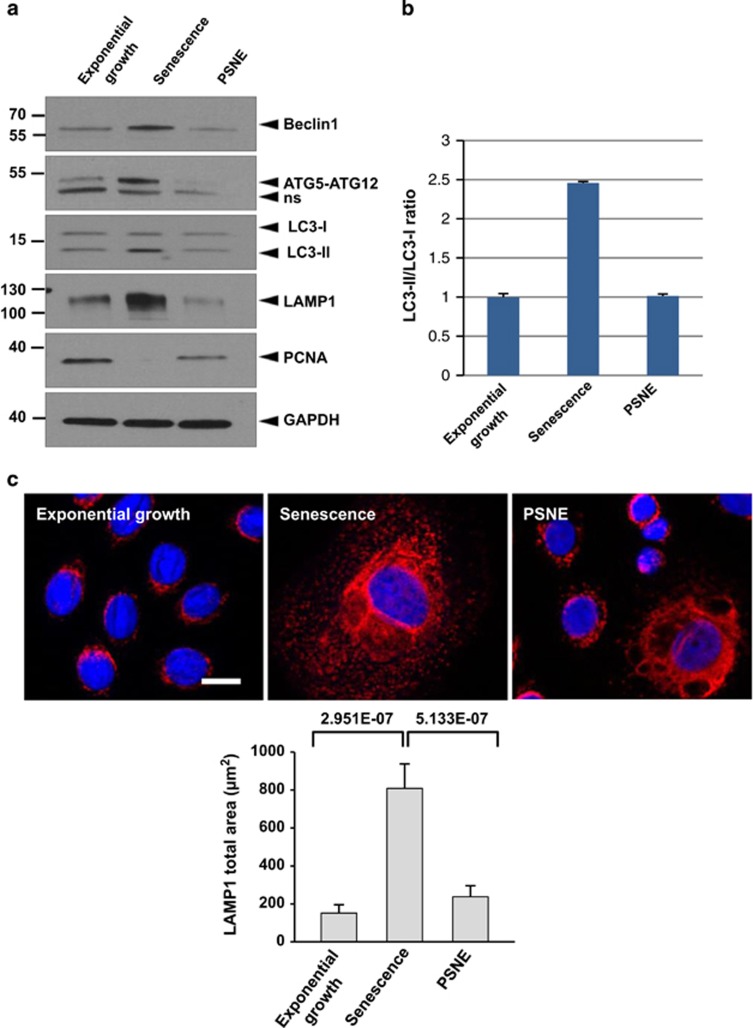
PSNE cells display less macroautophagic markers than their senescent progenitors. (**a**) Western blotting analysis of Beclin-1, ATG5, LC3 and LAMP-1 in protein extracts from NHEKs K18FC at the exponential growth phase, at the senescent plateau and at PSNE. PCNA was used as a proliferation marker, and GAPDH (glyceraldehyde 3-phosphate dehydrogenase) as a loading control. The anti-ATG5 antibody reveals the covalent ATG5–ATG12 complex. ns: non-specific band. (**b**) Band intensity of LC3 I and LC3II were quantified, and the LC3II/I ratios are given. Results are normalized to the value obtained in exponentially growing NHEKs. Results are representative of three independent experiments performed on two different donors. (**c**) LAMP-1 immunofluorescence assays performed on the same cells. Upper panels: Representative Apotome microscopic images. Bar represents 20 *μ*m. Lower panel: LAMP1-staining area was quantified with ImageJ. Measures were done in five independent microscopic fields for a total of at least 100 cells for each case. The histogram represents the average±S.D. of five counts. Results are representative of at least two experiments performed on two different donors

In order to determine whether PSNE involves escape of autophagic programmed cell death, we investigated the level of macroautophagy in PSNE cells compared with their senescent progenitors. The expression of several autophagic markers was examined by western blotting. The expression of ATG6/Beclin-1 increased at senescence compared with exponentially growing cells and returned to basal level in PSNE cells (Figure 2a). The formation of the ATG5–ATG12 complex increased at senescence and decreased again in PSNE cells (Figure 2a). Accordingly, the ratio between the cleaved and lipidated form of LC3 (LC3II) on the immature form (LC3I) increased at senescence and decreased again in PSNE cells (Figure 2a). LAMP-1, a marker of autolysosomes and lysosomes,^36^ displayed a similar expression pattern, i.e., increase at senescence and decay in the population of emergent cells (Figure 2a). An immunofluorescence staining of LAMP-1 confirmed that in PSNE cells the mass and density of lysosomes and autolysosomes is strongly reduced compared with senescent cells (Figure 2b). Taken together, these results suggest that PSNE cells have lost the high and lethal macroautophagic activity of their senescent progenitors. The loss of autophagic activity occurs at all stages of the process, from initiation to final stages.

### The level of macroautophagy dictates the outcome of senescent cells

**Figure 3:**
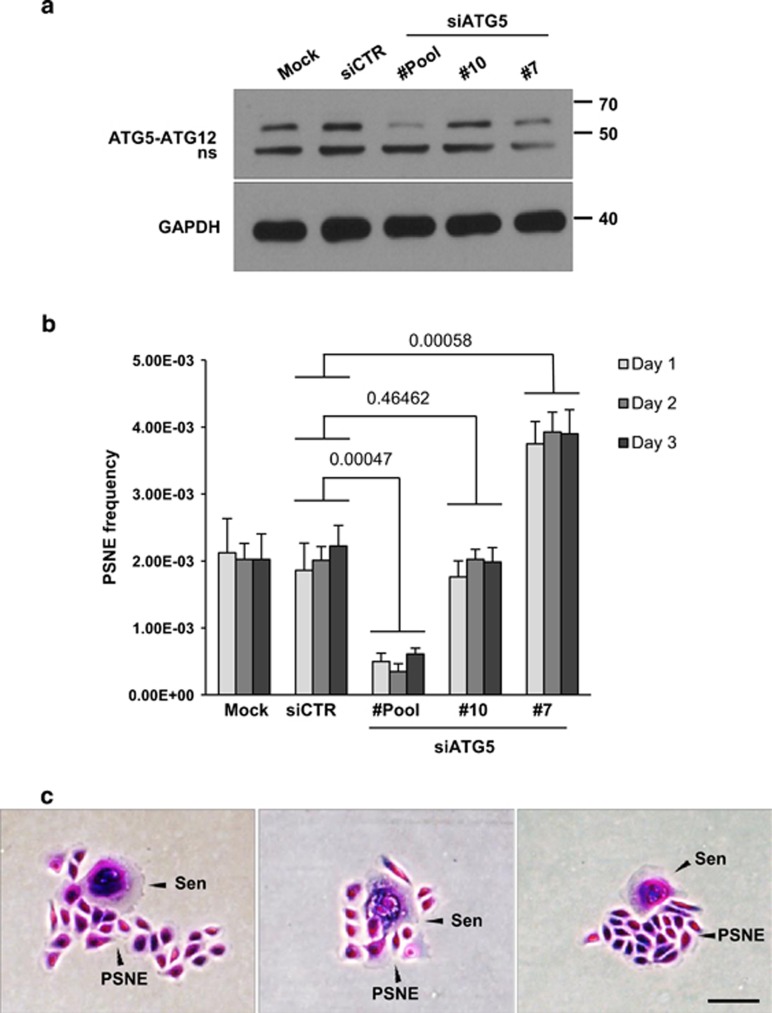
Invalidating *atg5* in H_2_O_2_-induced premature senescent NHEKs favors or inhibits PSNE according to the efficacy of siRNAs. H_2_O_2_-induced premature senescent NHEKs K18FC were transfected with a pool of four siRNAs targeting *atg*5 (#pool), two individual siRNAs (#10 and #7) targeting *atg*5, a pool of non-target siRNAs (siCRT) or treated only with the transfectant (Mock). (**a**) Verification of the efficacy of siRNAs by western blotting performed 48 h posttransfection. The anti-ATG5 antibody reveals the covalent ATG5–ATG12 complex. ns: non-specific band. (**b**) Twenty-four hours posttransfection, cells were seeded at low density, and PSNE frequency was measured at day 1, 2 or 3 after seeding. The counts were performed in eight independent culture dishes. The given results are the mean±S.D. of all counts. *P*-values calculated using the Student's *t*-tests are given. This experiment is representative of three independent ones. (**c**) Representative images of PSNE clones after fixation and coloration with crystal violet. Sen points a senescent cell, and PSNE the clone of emergent cells. Bar represents 10 *μ*m

To continue to address the question of whether the generation of PSNE cells needs escaping of autophagic cell death, we inhibited macroautophagy in senescent cells and examined the impact on PSNE. NHEKs were induced in premature senescence by a sublethal H_2_O_2_ treatment as previously described.^18^ Two batches of such H_2_O_2_-induced senescent cells were transfected with a pool of four control small interfering RNAs (siRNAs), a pool of four siRNAs targeting *atg5* or two different single siRNAs targeting *atg5*. A batch of cells was used 48 h posttransfection to check the efficacy of siRNAs on the formation of the ATG5–ATG12 complex (Figure 3a). Another batch of cells was plated at low density and monitored for PSNE. Surprisingly, the different siRNAs gave opposite results: cells in which *atg5* was very efficiently invalidated produced only very few PSNE clones, whereas those in which *atg5* was only partially affected underwent PSNE with a frequency about twofold higher than that of control cells (Figures 3b and c).

**Figure 4:**
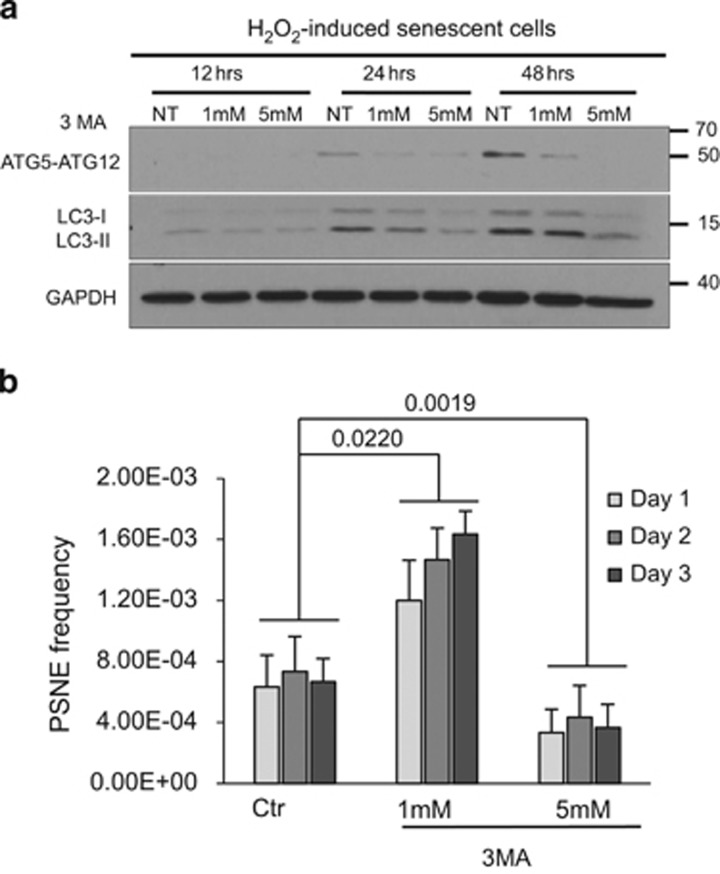
Lowering macroautophagy using 3-MA in H_2_O_2_-induced premature senescent NHEKs favors or limits PSNE depending on the inhibitor concentration. (**a**) Exponentially growing NHEKs K1MC were treated by H_2_O_2_ to induce premature senescence. They were then treated by 3-MA or its diluent H_2_O (NT), and the proteins were extracted at different times after the beginning of the treatment. The activation of ATG5 in ATG5–ATG12 covalent complex and of LC3 in LC3-II was analyzed by western blotting. (**b**) Exponentially growing NHEKs K18FC were treated by H_2_O_2_ to induce premature senescence. They were then treated by 3-MA or its diluent H_2_O, seeded at low density and monitored for emergence frequency. The counts of emerging clones were performed in four independent culture dishes at days 1, 2 and 3 postseeding. The given results are the mean±S.D. of all counts. *P*-values calculated using the Student's *t*-tests are given. The results are representative of five independent experiments

To further challenge this potential dose effect of autophagy inhibition on PSNE, we inhibited macroautophagy with 3-methyl adenine (3-MA), which blocks the activity of hVps34.^37^ We used 3-MA at two concentrations, 5 mM, a concentration classically used, and a much lower concentration, 1 mM. We verified that the two concentrations have a dose effect on the formation of the ATG5–ATG12 complex and on the lipidation of LC3 (Figure 4a). We applied 3-MA at these two concentrations on H_2_O_2_-induced senescent cells. One millimolar 3-MA significantly increased the emergence frequency, whereas 5 mM slightly decreased it (Figure 4b).

**Figure 5:**
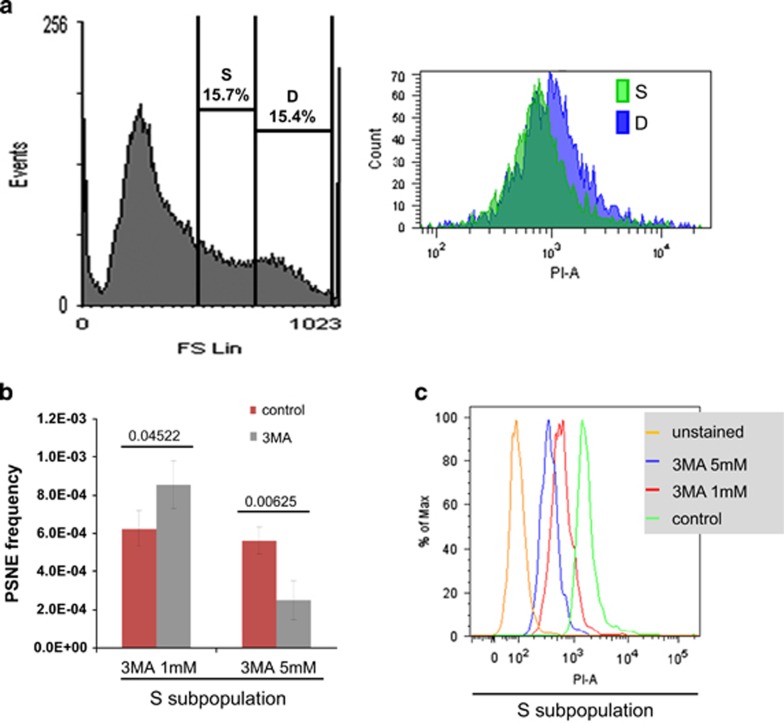
Inhibiting macroautophagy in senescent NHEKs favors or limits PSNE depending on the rate of inhibition. (**a**) NHEKs 13.20 were taken at the beginning of the senescent plateau and analyzed by flow cytometry as a function of forward scatter factor value (left histogram, the *x* axis represents forward scatter factor value). The delineation of S and D subpopulations is indicated. Cells of the S and D subpopulations were characterized by PI staining (right histogram; the *x* axis represents the PI fluorescence intensity (PI-A)). (**b**) Cells of the S subpopulation were sorted, seeded at low density, treated with 3-MA or its diluent H_2_O and monitored for their ability to generate PSNE clones. The counts of emerging clones were performed in eight independent culture dishes. The given results are the mean±S.D. of all counts. *P*-values calculated using the Student's *t*-tests are given. These experiments are representative of two independent ones. (**c**) NHEKs 13.20 were taken at the beginning of the senescent plateau, treated with 3-MA or its diluent H_2_O, stained by PI and analyzed by flow cytometry. The given plots of PI intensity (PI-A) were extracted using FlowJo and concern only the S subpopulation

We next wanted to enlarge these results to normal senescence. In a previous study,^10^ we had demonstrated that among senescent NHEKs the subpopulation of the 15% of cells with the largest size and highest granularity is the one that has the highest mortality index (subpopulation D). The subpopulation of the 15% of cells with size and granularity values just below is composed of fully senescent but still alive cells (subpopulation S). Here we sorted the S and D subpopulations by flow cytometry (Figure 5a). A batch of each subpopulation was stained with propidium iodide (PI) to check their viability and confirm their status (Figure 5a). An unstained batch of cells from the subpopulation S was seeded at low density, treated by 3-MA and monitored for PSNE. Again, 1 mM 3-MA significantly increased the emergence frequency, whereas 5 mM significantly decreased it (Figure 5b).

**Figure 6:**
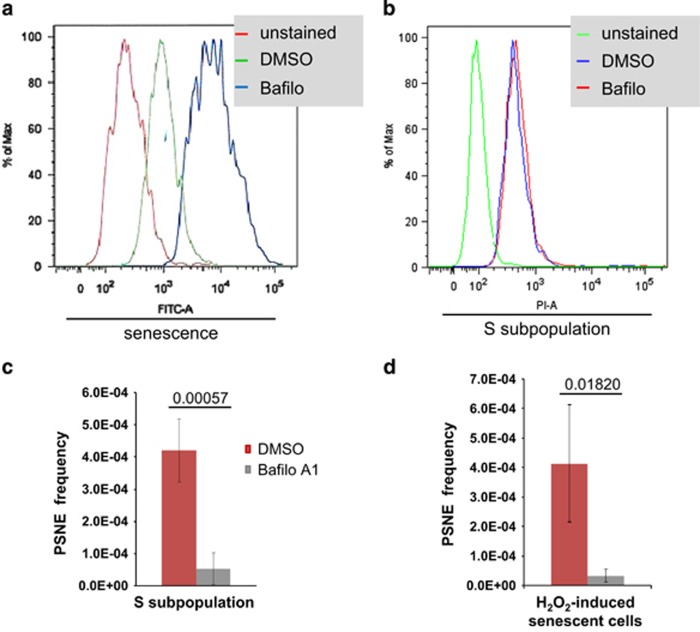
Maintaining an autophagic flux is indispensable for postsenescence emergence. NHEKs 13.20 were taken at the beginning of the senescent plateau, treated with Bafilomycin A1 or its diluent dimethyl sulfoxide (DMSO), stained by Lysotacker or PI and analyzed by flow cytometry. (**a**) Verification of the efficacy of Bafilomycin A1 on the autophagic activity of senescent NHEKs. The given values of Lysotracker intensity (FITC-A) were extracted using FlowJo. (**b**) Analysis of the effect of Bafilomycin A1 on the viability of senescent NHEKs. The given values of PI intensity (PI-A) were extracted using FlowJo and concern only the S subpopulation. (**c**) Emergence frequency of senescent NHEKs 13.20 treated by Bafilomycin A1 or its diluent DMSO. The counts of PSNE clones were performed in 4–8 independent culture dishes. The given results are the mean±S.D. of all counts. *P*-values calculated using the Student's *t*-tests are given. These experiments are representative of two independent ones. (**d**) Emergence frequency of H_2_O_2_-induced premature senescent NHEKs 13.20 treated by Bafilomycin A1 or its diluent DMSO. The counts of PSNE clones were performed in 4–8 independent culture dishes. The given results are the mean±S.D. of all counts. *P*-values calculated using the Student's *t*-tests are given. These experiments are representative of four independent ones

**Figure 7:**
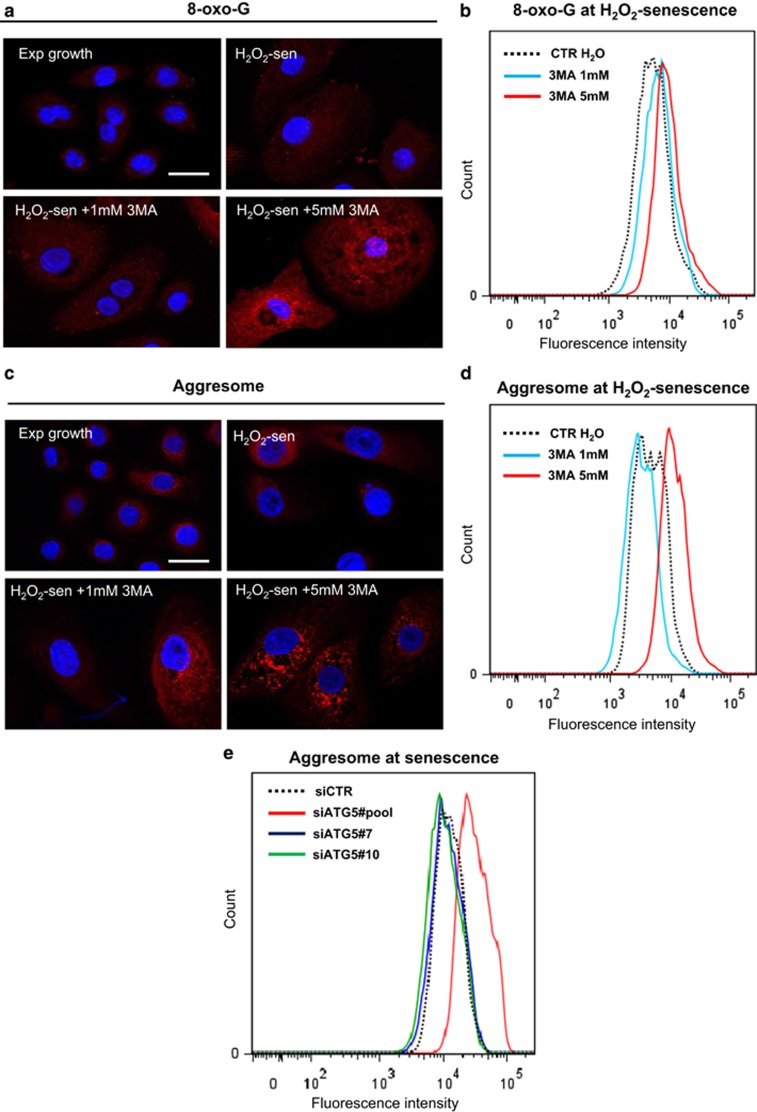
Senescent NHEKs invalidated for *atg*5 or treated by 3-MA accumulate altered components in a dose-dependent manner. (**a**) Representative Apotome microscopic images of 8-oxo-G in exponentially growing and H_2_O_2_-induced premature senescent NHEKs K1MC treated or not for 48 h with 1 or 5 mM 3-MA. Bar represents 20 *μ*m. (**b**) Quantitative detection of 8-oxo-G by flow cytometry in H_2_O_2_-induced premature senescent NHEKs K1MC treated or not for 48 h with 1 or 5 mM 3-MA. (**c**) Representative Apotome microscopic images of aggresomes in the same cells as in panel **a**. Bar represents 20 *μ*m. (**d**) Quantitative detection of aggresomes by flow cytometry in the same cells as in panel **b**. (**e**) Quantitative detection of aggresomes by flow cytometry in cells at the senescence plateau invalidated for *atg*5 as in Figure 3

A possible interpretation of all these results would be that reducing only partially the macroautophagy would allow escaping cell death and continue to ensure the quality control indispensable to the resumption of cell cycle; drastically reducing the macroautophagic level could as well allow cell death to escape but could impair the quality control carried out by autophagy and hence impair the cell ability to undergo mitosis. To challenge this interpretation, we performed three experiments. First, we measured the level of cell death induced by the two concentrations of 3-MA by a PI staining assay. The results confirm that 3-MA infers a reduction in cell death rate, with an only slight dose effect (Figure 5c). Second, we used Bafilomycin A1, which blocks the macroautophagic flux by inhibiting the latest phases of the process (inhibits the fusion of autophagosomes with lysosomes and the activity of H^+^ pumps.^38, 39^ We checked the efficiency of Bafilomycin A1 by a Lysotracker staining (Figure 6a). We also checked that Bafilomycin A1 did not change the death rate of NHEKs by a PI staining (Figure 6b). We then applied Bafilomycin A1 to senescent cells of the S subpopulation and to H_2_O_2_-induced senescent cells. In both cases, this resulted in an almost complete abolition of PSNE (Figure 6c), confirming that maintaining an autophagic flux is indispensable for the occurrence of PSNE. Third, we evaluated the quantity of damaged components in senescent cells where macroautophagy was inhibited. We examined 8-oxo-7-hydroxyguanosine (8-oxo-G) by immunofluorescence and flow cytometry. The results clearly show that 3-MA accentuates in a dose-dependent manner the accumulation of 8-oxo-G in senescent cells (Figures 7a and b). We also examined the formation of aggresomes of denatured proteins. They were clearly increased in senescent NHEKs treated by 5 mM 3-MA or by the very efficient pool of siRNAs targeting *atg*5, whereas they were found at a level almost similar to that of control cells in cells treated by 1 mM 3-MA or with the poorly efficient individual siRNAs (Figures 7c–e).

Taken together, these results suggest that, to be able to generate neoplastic emergent cells, senescent cells must have a macroautophagic level lower enough to escape cell death but higher enough to ensure a minimal quality control.

### The progenitors of PSNE cells display a moderate autophagic activity and a moderate oxidative stress

**Figure 8:**
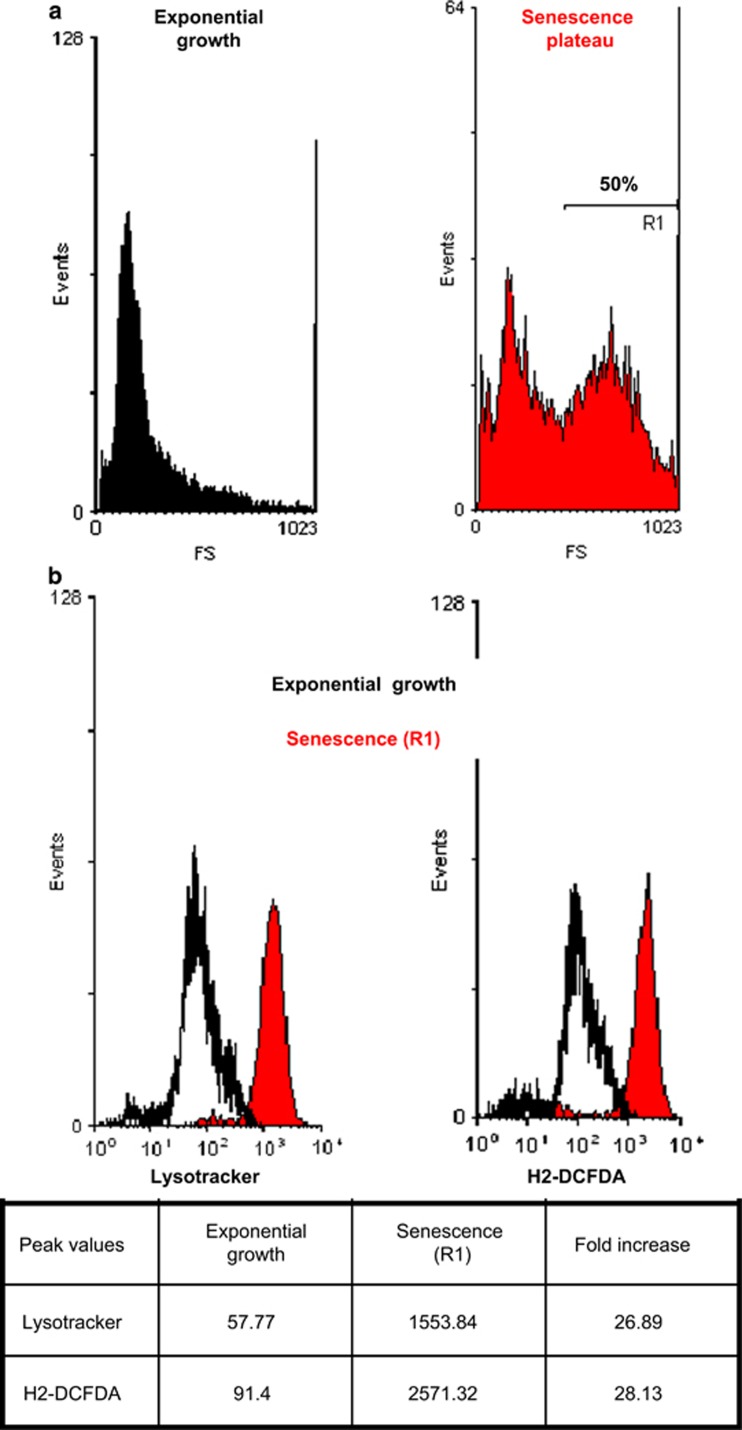
Autophagic activity and steady-state levels of ROS in exponentially growing *versus* senescent NHEKs. NHEKs 13.20 at the exponential growth phase (black) and senescence plateau (red) were stained with Lysotracker and H2-DCFDA and analyzed by flow cytometry. (**a**) Forward scatter (FS) factor analysis. The population at the senescence plateau shows two main peaks of size; the first one corresponds to residual small growing cells; the second one (R1) corresponds to senescent cells, including the S1, S2, D1 and D2 subpopulations of Figure 9. (**b**) Histograms of Lysotracker (left panel) and H2-DCFDA (right panel) staining intensities of exponentially growing (dark) and R1 senescent NHEKs (red). Peak values of the Lysotracker and H2-DCFDA stainings are given

**Figure 9:**
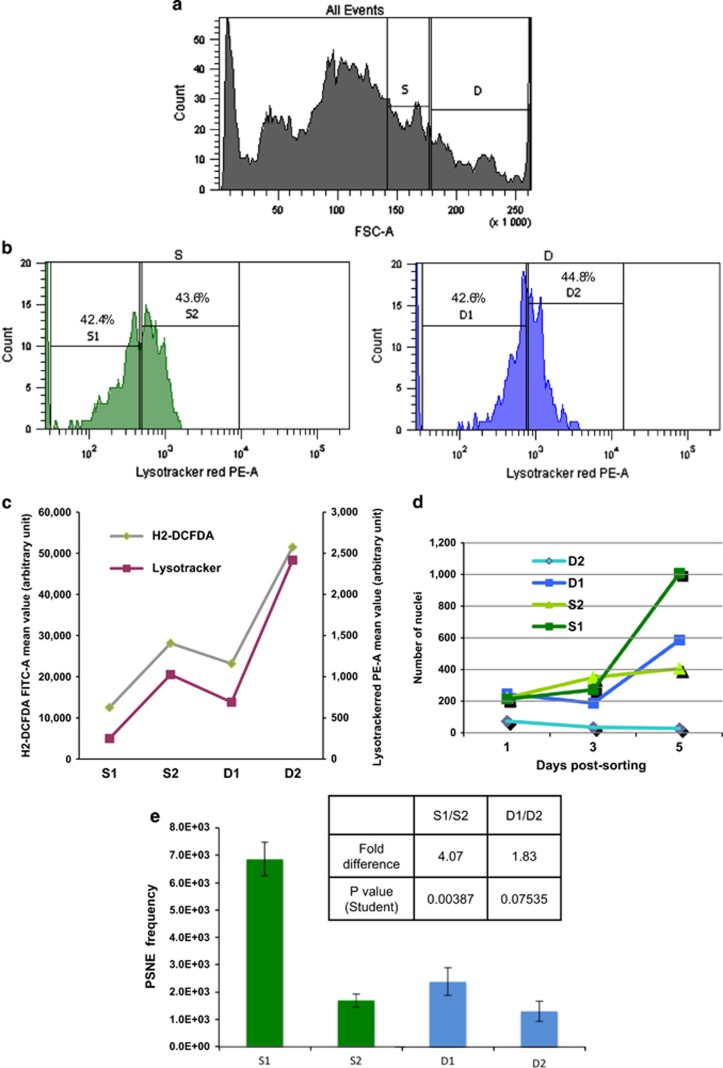
The probability of senescent cells to generate PSNE clones is linked to their macroautophagy and ROS levels. (**a**) Flow cytometric histogram of NHEKs 13.20 at the senescence plateau according to forward scatter factor (FSC-A) and showing the S and D subpopulations. (**b**) Flow cytometric histograms of the S (in green) and D (in blue) senescent subpopulations according to their Lysotracker staining intensity and showing the S1, S2, D1 and D2 subpopulations. (**c**) Mean values of the Lysotracker and H2-DCFDA staining intensities of the four subpopulations. (**d**) The four subpopulations were seeded in four-well plates and, every 24 h, fixed, stained with Hoechst and automatically counted as described in Materials and Methods section. (**e**) The four subpopulations were seeded at low density and monitored for PSNE. The counts of clones were performed in four independent culture dishes. The given results are the mean±S.D. of all counts. The indicated fold difference corresponds to the ratio of the means of S1 on S2 and D1 on D2. *P*-values were calculated using the Student's *t*-tests

To further confirm or infirm the above conclusion, we investigated the ability of senescent NHEKs to generate PSNE clones as a function of their level of macroautophagic activity and, as this activity is induced by the accumulation of oxidative damages,^21^ as a function of their steady-state level of ROS. First, we measured the level of ROS and the level of macroautophagy in the overall senescent NHEK population in comparison with exponentially growing cells. ROS concentration was measured with 2',7'-dichlorodihydrofluorescein diacetate (H_2_-DCFDA), a fluorescent H_2_O_2_ sensor. The results indicate that ROS and macroautophagy levels are, respectively, 26 and 28 times higher at senescence than during exponential growth (Figure 8). Second, the senescent population was divided in subpopulations S and D as above (Figure 9a). Then each S and D subpopulation was divided again according to their Lysotracker staining into two new subpopulations named S1, S2, D1 and D2 (Figure 9b). Interestingly, the H_2_-DCFDA staining of the four subpopulations exactly paralleled the Lysotracker staining (Figure 9c), showing that the level of macroautophagy is linked to the level of ROS. In parallel, cells of the four subpopulations were sorted and seeded in four-well plates. Each 24 h, cells were fixed, stained with Hoechst and automatically counted under a fluorescent microscope. The results indicate that the D2 subpopulation declined progressively and never generated PSNE cells. The S2 subpopulation did not divide but remained alive without generating PSNE cells. In contrast, the D1 and S1 subpopulations generated PSNE cells (Figure 9d). Other batches of cells of the four subpopulations were seeded at low density and monitored for PSNE in order to precisely measure the PSNE frequency. It is the subpopulation S1 that generated the highest clone number (Figure 9e). Taken together, these results indicate that the progenitors of PSNE cells are found among the senescent viable cells, which display a moderate steady-state ROS level and an ensuing moderate macroautophagy level, compared with the overall population at the senescence plateau.

### The level of oxidative stress of the senescent progenitors of PSNE cells is determined by the level of activation of the NF-*κ*B/MnSOD axis

**Figure 10:**
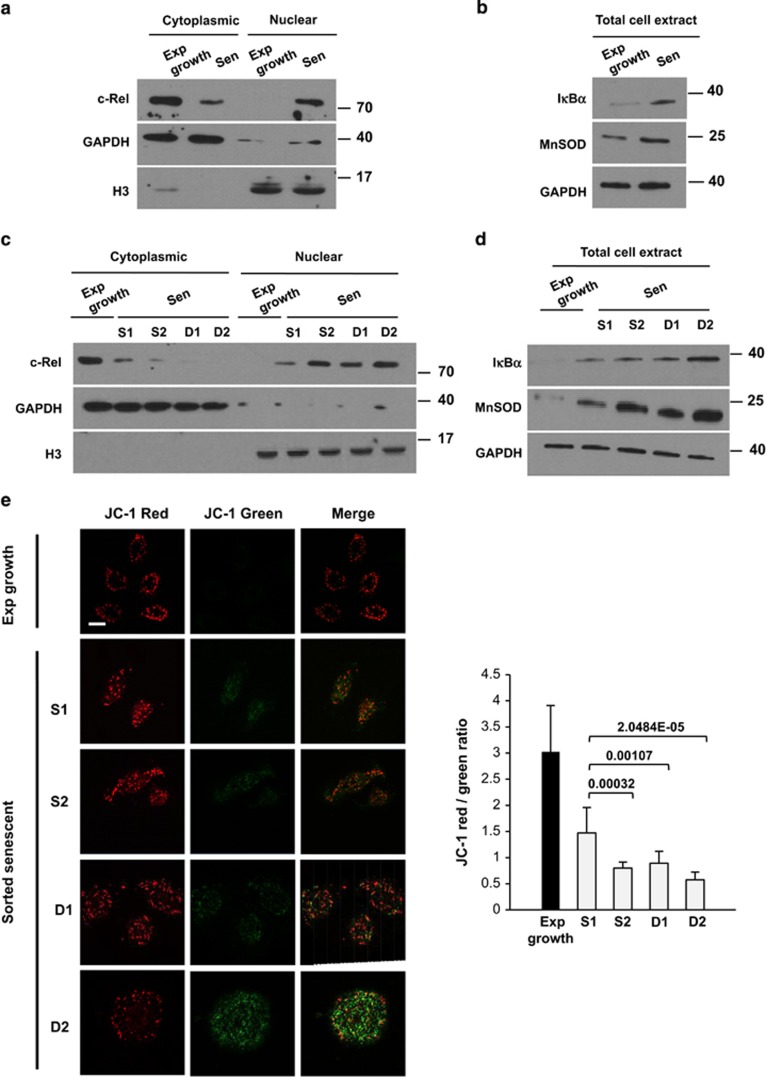
Gradation of NF-*κ*B activation and mitochondrial dysfunction in senescent NHEKs. (**a** and **b**) Western blotting analysis of cRel, I*κ*B*α* and MnSOD in cytoplasmic and nuclear extracts or in total cell extracts of NHEKs K18FC at the exponential growth phase and at the senescent plateau. (**c** and **d**) S1, S2, D1 and D2 subpopulations of senescent NHEKs K18FC were sorted as in Figure 9, and western blotting analysis of cRel, I*κ*B*α* and MnSOD was performed. GAPDH (glyceraldehyde 3-phosphate dehydrogenase) and histone H3 were used as marker and loading control for cytoplasmic and nuclear extracts, respectively. (**e**) Left panel: Representative confocal microscopic images of JC-1 staining assays performed in cells as in panels (**c** and **d**). Bar represents 20 *μ*m. Right panel: quantification of the red/green ratio using ImageJ. The counts were performed in >40 cells. The given results are the mean±S.D. of all counts. *P*-values were calculated using the Student's *t*-tests

We previously established that a NF-*κ*B/MnSOD/H_2_O_2_ pro-oxidant pathway is activated at senescence in NHEKs, producing oxidative damages to nucleus and mitochondria and therefore inducing autophagic cell death.^18, 21^ Here we wanted to determine whether the level of H_2_O_2_ in the different senescent subpopulations is dictated by the degree of activation of the NF-*κ*B/MnSOD axis. To assay the activation of NF-*κ*B transcription factors, we examined the nucleocytoplasmic translocation of cRel, a member of the family, and the activation of I*κ*B*α*, one of its target genes. The results show that at senescence cRel is translocated into the nucleus and I*κ*B*α* is upregulated. MnSOD (SOD2), the mitochondrial redox enzyme, is also upregulated (Figures 10a and b), confirming our previous data. Interestingly, the examination of the level of cRel activation and MnSOD upregulation in the four subpopulations revealed a range of activation from S1 to D2, with the lowest level of activation in S1 and the highest in D2 (Figures 10c and d). Therefore, the mild level of oxidative stress in the S1 subpopulation is the direct consequence of a mild activation of the NF-*κ*B/MnSOD axis.

As MnSOD is a mitochondrial enzyme, the H_2_O_2_ overproduced following its upregulation should primarily affect mitochondria. We therefore evaluated mitochondrial fitness by measuring mitochondrial membrane potential. Young and senescent NHEKs of the four subpopulations were stained with the JC-1 cationic dye, which accumulates in mitochondria in a potential-dependent manner and whose fluorescence shifts from red to green with mitochondrial depolarization. We found that the red/green ratio was significantly higher in S1 than in D2 cells, indicating that the mitochondria were less damaged and more functional in S1 than in D2 cells (Figure 10e). Therefore there is in the senescent population a strict correlation between the level of oxidative stress, the level of activation of the NF-*κ*B/MnSOD axis, the ratio of functional/dysfunctional mitochondria, the level of autophagic activity and the ability to generate PSNE clones.

## Discussion

Macroautophagy is activated at senescence. This was shown for normal senescent fibroblasts,^40^ IMR90 fibroblasts overexpressing H-RasV12,^11^ long-term cultured and repeatedly stimulated T lymphocytes,^41^ normal senescent epidermal keratinocytes,^10^ normal biliary epithelial cells^42^ and some cancerous cell lines re-induced in senescence upon various drug treatments.^43, 44^ This opens the question of what are the roles and consequences of the macroautophagic activity of senescent cells. Here we show that this senescence-associated macroautophagy determines the outcome of senescent keratinocytes, depending on its level of activation.

The outcome of senescent cells can differ from one cell type to another. In contrast to senescent fibroblasts that are irreversibly cell cycle arrested,^14^ senescent NHEKs, as well as senescent HMECs, either die or re-enter mitosis to generate postsenescent emergent cells that display neoplastic properties.^22, 23^ We demonstrate here that the senescent NHEKs, the most prone to generate PSNE cells, are those displaying a moderate autophagic activity. We understand by moderate activity an activity 2–5-fold lower than that displayed by the average of senescent cells (Figure 8c) but >20 fold higher than that displayed by young proliferating cells (Figure 7). The level of autophagic activity in senescent cells is strictly correlated with their ROS level, which is itself directly correlated to the level of activation of the NF-*κ*B/MnSOD pro-oxidant pathway and to the fitness of the mitochondria population. When moderate, the macroautophagic activity enables senescent NHEKs (i) to escape autophagic programmed cell death and (ii) to ensure the elimination of various accumulated altered components, especially the oxidized ones, which could be deleterious. That way, senescent cells can survive and, for some of them, re-enter mitosis and generate daughter cells, which should be themselves enough clean to survive and proliferate. Macroautophagy thus has antagonistic roles in the outcome of senescent NHEKs. When overactivated, macroautophagy induces senescent cell death, hence reinforcing the tumor-suppressive role of senescence already assigned to its cell cycle arrest effect; when only moderately activated, macroautophagy favors senescent cell death escape and the new generation of neoplastic cells, hence contributing, in contrast, to a tumor-promoter role of senescence.

However, although escaping cell death and ensuring quality control are two necessary parameters of mitosis re-entry, they are not sufficient to explain the transformed and tumorigenic phenotype of PSNE cells, which necessarily involves genetic or epigenetic alterations. Previous work of our group had demonstrated that the NF-*κ*B/MnSOD/H_2_O_2_ pathway is not only responsible for NHEK senescence^18^ and the following autophagic cell death^21^ but also for the generation of PSNE cells in correlation with the acquisition of oxidative DNA damages.^22^ With the present report, it becomes clear that acquiring oxidative damages affecting genome integrity but keeping under control by autophagy those affecting other molecules and organelles could be the two key determinants of senescent cell outcome.

Data regarding the role of macroautophagy in carcinogenesis are the subject of controversial interpretations. Several studies suggest that macroautophagy could be activated in cancer cells under nutrient deprivation and hypoxia resulting from limited angiogenesis and help cancer cells to survive.^45^ In that sense, macroautophagy can be viewed as tumor promoter. In contrast, it was shown that several human cancers harbor inactivating mutations or deletions in several *atg* genes, including *atg67/beclin-1*,^46^ its partner *UVRAG*,^47^ as well as *atg2B*, *atg5* and *atg9B*,^48^ what defines them as tumor-suppressor genes. However, at least regarding *atg6/beclin-*1 and *UVRAG*, the reported mutations were always monoallelic deletions. Moreover, mice invalidated for *beclin1* on both alleles were non-viable, whereas heterozygous *beclin1*+/− mice were shown to spontaneously develop preneoplastic or malignant lesions with age.^49, 50^ This suggests that, in accordance with our present *in vitro* data, the consequence of a macroautophagy defect is dose-dependent. Very interestingly, it was shown that *beclin1*+/− mammary epithelial cells display more DNA damages than *beclin1*+/+ cells when subjected to metabolic stress,^51^ suggesting that a moderate macroautophagic activity may promote tumorigenesis by keeping alive cells with genomic alterations.

## Materials and Methods

### Cell culture, SA-*β*-Gal assay, Trypan blue exclusion assay

NHEKs were purchased from Clonetics (Basel, Switzerland; CC-2501) or Tebu-bio (Le Perray en Yvelines, France; 102.05a). We used cells from five different donors of different sex, race and age (referred to as 4F0315, 2F1958, 13.20, K1MC, K18FC). Cells were obtained anonymously, and informed consent of each skin donor was obtained by the supplier. Cells were grown at 37 °C at the atmospheric O_2_ tension plus 5% CO_2_. The atmospheric O_2_ tension is nearly normoxic for cells from the epidermis.^52, 53^ Cells were cultured in the Epilife medium (MEPICF500, Invitrogen, Carlsbad, CA, USA) with 0.15 mM calcium supplemented with HKGS (S0015). Such a serum-free low-calcium medium was shown to minimize keratinocyte terminal differentiation.^54^ Cells were routinely seeded at 3500 cells/cm^2^ and always subcultured at 70% confluence. The number of population doublings (PDs) was calculated at each passage by means of the following equation: PD=log(number of collected cells/number of plated cells)/log2.

SA-*β*-Gal assays were performed as described in Dimri *et al.*^55^

For quantification of cell death by Trypan blue exclusion assays, NHEKs were incubated in 0.4% Trypan Blue solution for 5 min, and the cell suspension was loaded onto a Thoma counting chamber. Non-viable cells (blue) were counted under the microscope (Inverted microscope, Axiovert 40C, Carl Zeiss Microscopy, Jena, Germany).

### Induction of premature senescence by H_2_O_2_

NHEKs at the exponential growth phase were treated according to the donor by 20–50 *μ*M H_2_O_2_ every 24 h. The senescent phenotype (growth arrest, cell enlargement and acquisition of the SA-*β*-Gal marker) was clearly established in all cells after 48–72 h, as already published.^18^

### Inhibition of macroautophagy by siRNA or pharmacological inhibitors

SiRNAs were diluted in Lipofectamine RNAiMAX transfection reagent (Invitrogen) and incubated for 15 min at room temperature before being added to cells in fresh culture medium. Invalidation of atg5 was performed using 25 nM of a pool of four siRNAs (M-004374-04-0005, Dharmacon (Lafayette, LA, USA), Target sequences: 5′-GGAAUAUCCUGCAGAAGAA-3′—5′-CAUCUGAGCUACCCGGAUA-3′—5′-GACAAGAAGACAUUAGUGA-3′—5′-CAAUUGGUUUGCUAUUUGA-3′) or two different siRNAs from the on-target plus set of four siRNAs (LU-004374-00-0005, Dharmacon, Target sequences: #7: 5′-GGCAUUAUCCAAUUGGUUU-3′—#10: 5′-ACAAAGAUGUGCUUCGAGA-3′). A non-targeting siRNA pool (siGENOME RISC-Free Control siRNA, Dharmacon) was used as a control. Transfections were stopped after 24 h by renewing the culture medium.

3-MA (Sigma-Aldrich, St. Louis, MO, USA; M9281) and Bafilomycin A1 from *Streptomyces griseus* (B 1793), respectively, diluted in water and DMSO, were directly added to the culture medium at 1 or 5 mM for 3 MA and 5 nM for Bafilomycin and let to react for 48 h.

### Western blotting

For total protein extracts, cells were lysed in Hepes 27.5 mM pH 7.6, urea 1.1 M, NaCl 0.33 M, EGTA 0.5 mM, EDTA 2 mM, KCl 60 mM, DTT 1 mM and NP40 1.1%. For extracting cytoplasmic and nuclear proteins, the Subcellular Fractionation kit (Thermo Fischer Scientific Inc, Rockford, IL, USA; 78840) was used. The total protein concentrations were measured with the Bio-Rad protein assay (Bio-Rad, Hercules, CA, USA; 500-0006). Proteins were resolved by SDS-PAGE and transferred to PVDF membranes (Millipore, Bedford, MA, USA). Primary antibodies used were: GAPDH (Santa Cruz Biotechnology, Dallas, TX, USA; SC32233), PCNA (Dako Cytomation, Glostrup, Denmark; M0879), LAMP-1 (Santa Cruz Biotechnology SC17768), LC3 B (Cell Signaling, Boston, MA, USA; no. 2775), ATG5 (Cell Signaling no. 2630), Beclin-1 (Cell Signaling no. 3738), cRel (Santa Cruz no. sc-6955), I*κ*B*α* (Santa cruz, no. sc-1643), MnSOD (Calbiochem, Darmstadt, Germany; no. 574596), and histone H3 (Abcam, Cambridge, UK; no. 1791). Secondary antibodies used were peroxidase-conjugated anti-mouse IgG (Jackson Immunoresearch Laboratories, West Grove, PA, USA; 115-035-146), anti-rabbit (Jackson Immunoresearch Laboratories; 711-035-125) or anti-goat (Santa Cruz, no. sc-2033). Peroxidase activity was revealed using an ECL (enhanced chemiluminescence) or ECL advanced kit. The luminescence was captured with a Fuji intelligent dark box camera (LAS 3000, Fujifilm, Dusseldorf, Germany). Quantifications were performed with the Multigauge V3.0 software (Fujifilm).

### Immunofluorescence

For LAMP-1 detection, cells were fixed with 4% paraformaldehyde in PBS and permeabilized with 0.2% Triton-X100. For 8-oxo-G detection, cells were fixed in 4% paraformaldehyde in PBS and postfixed in 70, 95 and 99% methanol for 30 min at −20 °C and then rehydrated by 95 and 70% methanol at −20 °C for 3 min and three washes in PBS. The primary antibodies used were anti-LAMP-1 (Transduction Labs, Erembodegem, Belgium; L76620) and anti-8-oxo-G (AB5830 Chemicon International, Frederiksberg, Denmark). The secondary antibody used was Rhodamine RedX-conjugated anti-mouse IgG (Jackson ImmunoResearch Laboratories). Nuclei were stained by Hoechst 33258 at 50 ng/ml for 15 mn. Slides were analyzed with a Axio Imager Z1 microscope (Carl Zeiss Microscopy) equipped with an Apotome device enabling optical sectioning.

Aggresome detection was performed using the ProteoStat Aggresome detection kit (Enzo Life Sciences, Farmingdale, NY, USA) according to the manufacturer's instructions.

### Lysotracker and H_2_-DCFDA staining

Lysotracker and H_2_-DCFDA were purchased from Molecular Probes (Life Technologies, Thermo Fischer Scientific Inc). Living cells were incubated with probes directly added to the culture medium at 37 °C for 30 min as recommended by the supplier.

### Flow cytometric analysis and fluorescence-activating cell sorting

Flow cytometric analyses were performed using a Beckman Coulter (Pasadena, CA, USA) Epics XL-MCL, a BD LSR Fortessa (Becton Dickinson, Erembodegem, Belgium) or a BD FACSCanto II (Becton Dickinson). 8-oxo-G detection was performed exactly as above for microscopic analysis. Aggresome detection was performed using the ProteoStat Aggresome detection kit (Enzo life Sciences) according to the manufacturer's instructions. Collected data were exported to the WinMDI 2.9 (J. Trotter, Salk Institute for Biological Studies, La Jolla, CA, USA) or FlowJo (Ashland, OR, USA) softwares for detailed analysis. Sorting of NHEKs was performed on a BD FACS Aria (Becton Dickinson) or on a Coulter FACS Altra (Beckman Coulter). Selected subpopulations with the *ad hoc* forward scatter factor and/or fluorescent staining intensity values were electrostatically sorted in air, collected in complete culture medium and put again in culture.

### Follow-up of cell viability and proliferation after sorting

After sorting, cells were plated in four-well plates. Every 24 h, a plate was fixed with 4% paraformaldehyde in PBS, washed and stained by Hoechst 33258 at 50 ng/ml for 15 mn. Wells were then imaged using the 6 × 6 mosaic function of the Zeiss observer microscope (Carl Zeiss Micorscopy). The automatic counting of nuclei was performed using a Fiji's analysis template (Image J 137C, Bethesda, MD, USA), which permits to discriminate nuclei according to Hoechst-positive particle's size, intensity and circularity.

### Measure of PSNE frequency

Cells, either at the senescent plateau, sorted by FACS or H_2_O_2_-treated, were plated at 150 cells/cm^2^ in order to be completely isolated. Culture dishes were scrutinized every day for the appearance of PSNE clones. When having appeared at a sufficient number in control condition, dishes were fixed in ethanol 95% for 5 min, air dried, stained with crystal violet (1 mg/ml diluted in methanol 8%) for 10 min and washed with tap water. Clones were manually counted under a stereo microscope (Stemi 2000C, Carl Zeiss Microscopy). The sole clones taken into account were those well isolated and containing a senescent cell among emergent ones (Figure 3b) ensuring that they are *bona fide* PSNE clones originating from a senescent cell and not from some putatively contaminating young cells. The emergence frequency was then calculated by reporting the number of clones on the number of initially seeded senescent cells.

### Evaluation of mitochondrial health

Cells were plated in glass-bottom dishes (WillCo-dish-GWST 5040, WillCo wells BV, Amsterdam, The Netherlands). After a wash in PBS, cells were incubated with 1 μM of JC-1 dye (Life Technologies, T-3168, Thermo Fischer Scientific Inc) for 30 min and washed again. JC-1 staining was analyzed using a Zeiss confocal microscope (LSM 780, Carl Zeiss Microscopy). For recording the green fluorescence indicative of depolarization, cells were excited at 488 nm, and emission was detected using a 530±40 nm band pass filter. For recording the red fluorescence indicative of intact *Δψ*(m), cells were excited at 488 nm, and emission was detected using a 613±20 nm band pass filter.

## Abbreviations

UV: ultraviolet
ROS: reactive oxygen species
NHEK: normal human epidermal keratinocyte
HMEC: human mammary epithelial cells
PSNE: postsenescence neoplastic emergence
SA-*β*-Gal: senescence-associated-*β*-galatosidase activity
3-MA: 3-methyl adenine
PI: propidium iodide
8-oxo-G: 8-oxo-7-hydroxyguanosine
siRNA: small interfering RNA
H_2_-DCFDA: 2',7'-dichlorodihydrofluorescein diacetate
PD: population doubling
